# A specialty forged on its doubts

**DOI:** 10.1016/S1808-8694(15)31298-2

**Published:** 2015-10-20

All investigators, regardless of the academic area which they advocate for, are mainly motivated by the search for answers and solutions to intriguing questions within their area of performance and that contain in themselves positive possibilities for maintenance and survival of human species.

To reach such objectives, the link between questions and real life and understandable situations is fundamental. This link favors better understanding of topics and, if well planned, it ensures that the executed projects are more likely to provide useful answers to humanity. Otorhinolaryngology as a specialty has arisen from the attempt to answer specific questions, based on doubts generated from the needs of people dedicated to a thematic area, whose focus has probably changed in the past 3 centuries. Upon considering that a specialist has doubts only about what he/she knows, and the more knowledge gathered about an issue, the more complex are the questions posed, we can accept that a specialist is what he/she intends to answer, and their performance is defined by their doubts, questions and above all, their efforts to have them answered.

Getting to know the main intellectual uncertainties of professionals that work in the area and check whether there is a minimum standard preserved in any given geopolitical region may help us understand who we are as a specialty and what we want to become, in addition to showing us what to do, and maybe, what is being neglected. In spite of knowing that not everything that is studied is actually published and that, even when published, it is not always available, we understand that any investigator that intends to find reasonable answers to pertinent questions works hard to have his/her projects as part of indexed scientific periodicals that have some visibility.

**Chart. 1:**
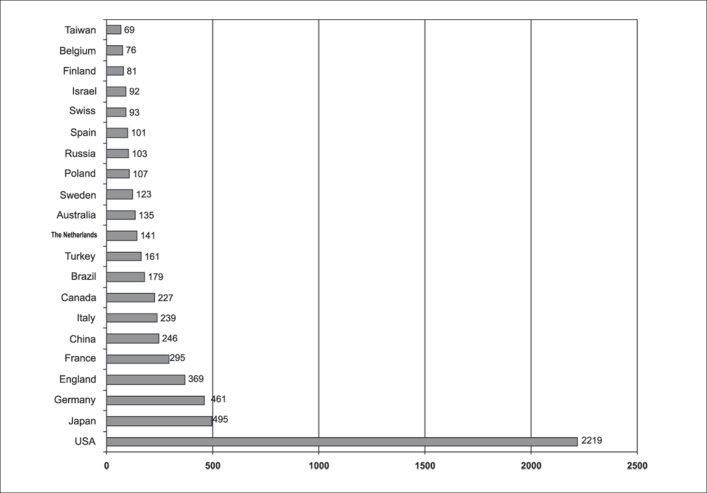
Distribution of number of scientific studies published and indexed by index medicus by country in years 2003-2005.

Based on such premises, we decided to investigate the product of world scientific publications and issues that are close to the essential topics of Otorhinolaryngology, be them basic or clinical aspects performed by any professional regardless of the training and including biases to human beings or other species. Within such perspective of indexation, visibility and academic diversity, we understand that the main available database would be the National Library of Medicine (NLM) which maintains index medicus for online access, covering over 9,000 titles of periodicals in the healthcare area. Using this database, we made a search based on anatomical region and whose key words were: Otolaryngology [Mj], Head and Neck Surgery [Mj], Ear (mesh), Nose (mesh), larynx (mesh), pharynx (mesh), neck (mesh), face (mesh), hearing (mesh), olfaction (mesh), gustation (mesh), speech (mesh), voice (mesh), swallow (mesh), articulation (mesh), learning (mesh) and breathing (mesh). The research project was designed to collect data from the past 5 years and it produced a total of 24,389 articles that are being analyzed concerning contents, authors, authors' affiliation, and country in which it was conducted. Data on years 2003, 2004 and 2005 are the main focus of our presentation, amounting to 6,427 articles from identifiable 78 countries and 274 other articles. Data relative to distribution by country is shown in Chart 1.

As expected, we could notice that American production amounts to 32.3% of the whole Medline indexed production, followed by about 20 countries with regular production, special highlight to the group of the key most powerful countries, plus Brazil and Turkey.

We noticed that countries with relevant specialty journals indexed in Medline did, as expected, have a greater share in the general context. Given that RBORL has been part of the database for only 3 months, Brazil could not be given credit considering that all other 15 main countries listed in Chart 1 had had at least one indexed periodical. Just for the sake of forecast, if we add up all articles published in RBORL in years 2003, 2004 and 2005 we would come to a total of 423 studies that would place us behind only the United States, Germany and Japan.

**Chart 2:**
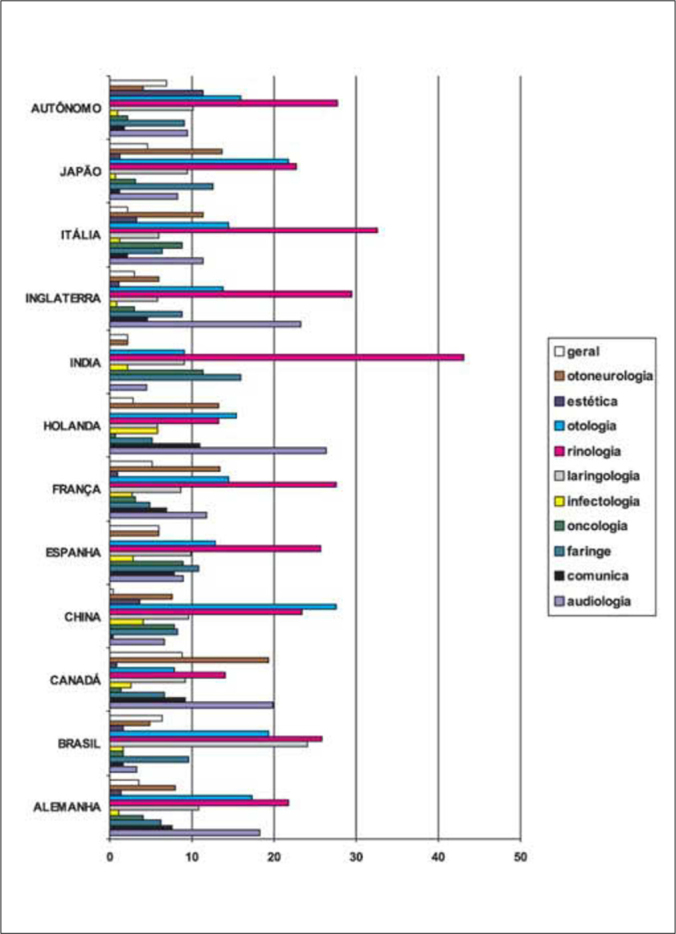
Distribution of subject concentration areas in articles published according to authors' country of origin 2001-2005.

**Chart 3:**
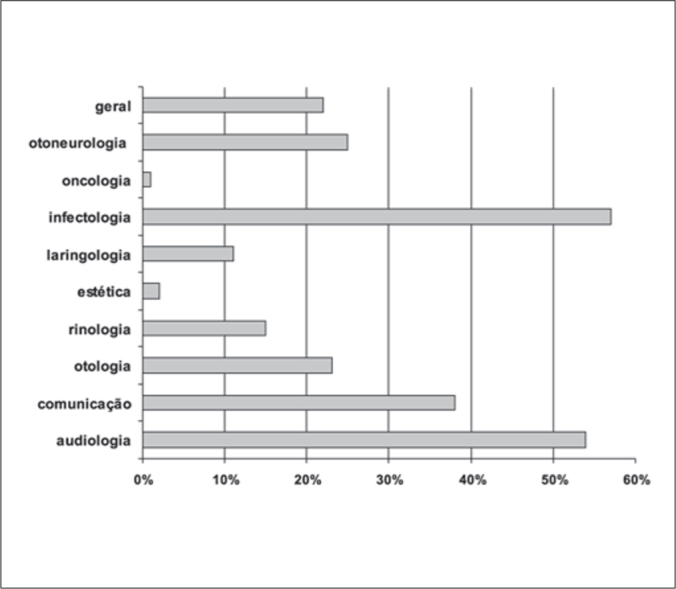
Distribution of papers with pediatric bias in the subject concentration area 2001-2005.

In keeping with the objective of the survey, we tried to locate the most important areas of investigative concentration of the articles collected, identifying the following ones: audiology, communication, otology, rhinology, facial esthetical surgery, oncology, infectology, otoneurology, laryngology and general otorhinolaryngology. We did not deem adequate to separate pediatric and adult areas at first, but we did separate the two focuses in each one of the areas of concentration and the results are presented in charts 2 and 3.

We noticed that each country shows its own trend, with greater emphasis on one subject or another. This may be due to the specific character of the journals available in different countries.

As a whole, we noticed that rhinology is one of the most prevalent areas in all regions, followed by otology/audiology. The number of questions specifically related to childhood in the general pool of questions shows that the distribution of the different areas is relatively non-uniform.

As to specific issues within each area of thematic concentration, it was surprising to see the number of articles whose main interest was the physiology of sensorial organs. The ones that predominated were hearing, olfaction and balance.

As we can see, data are still being compiled and are currently raw, but they have a huge potential.

Best regards,

Henrique Olival Costa

